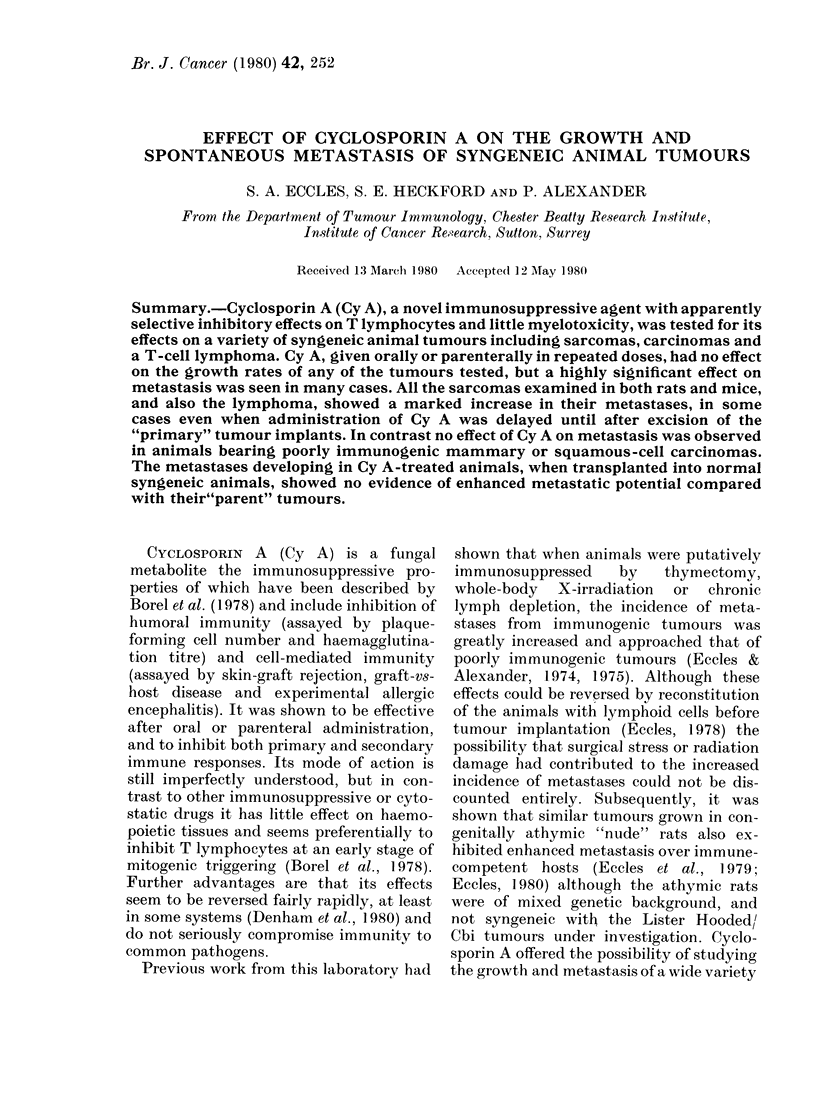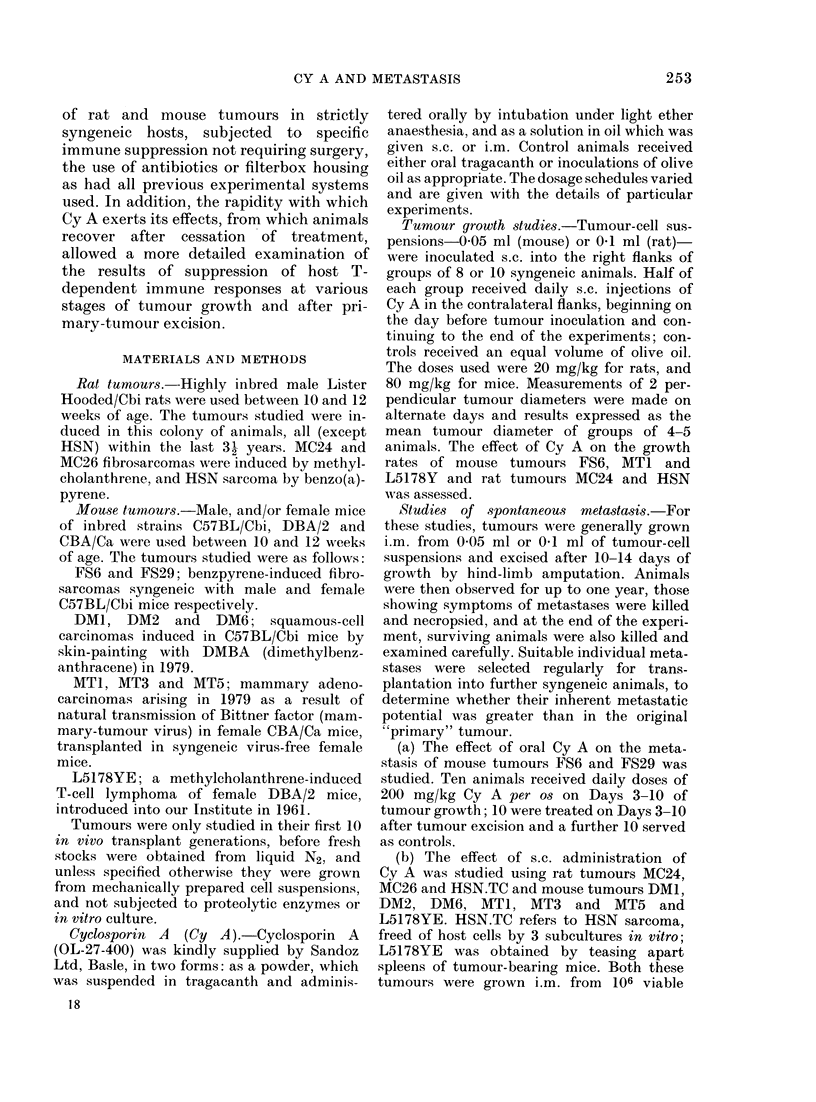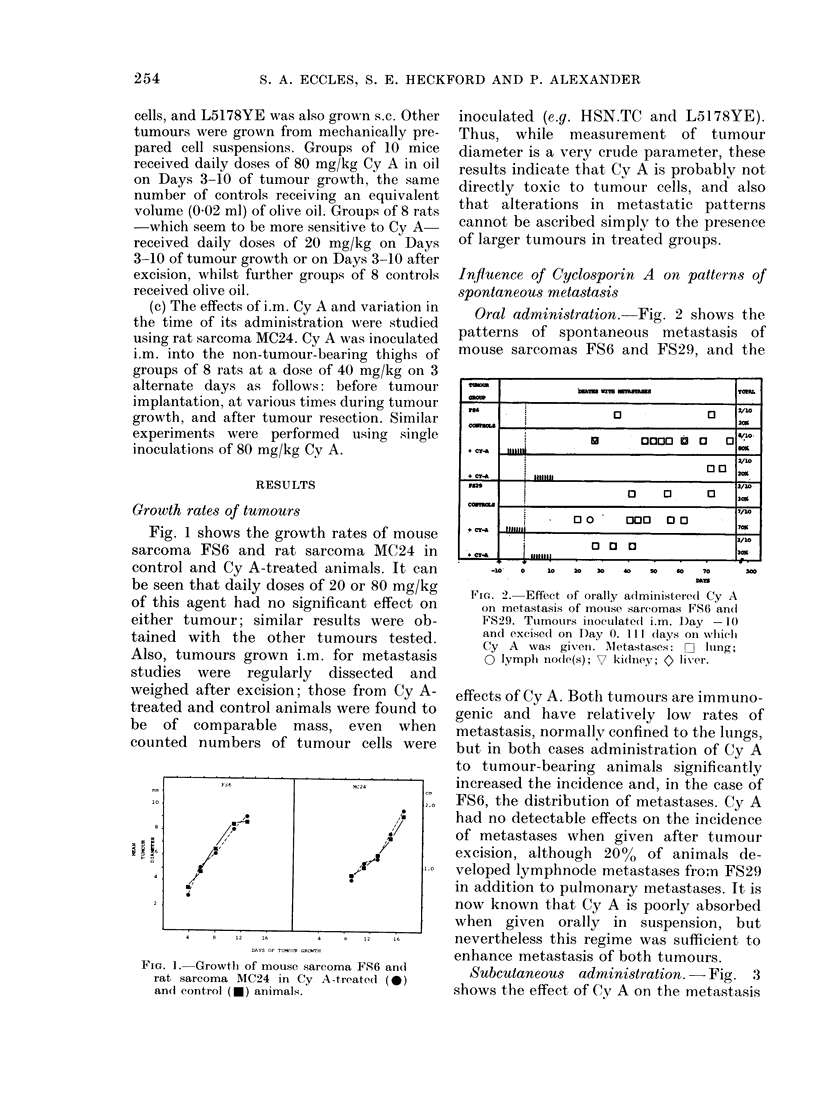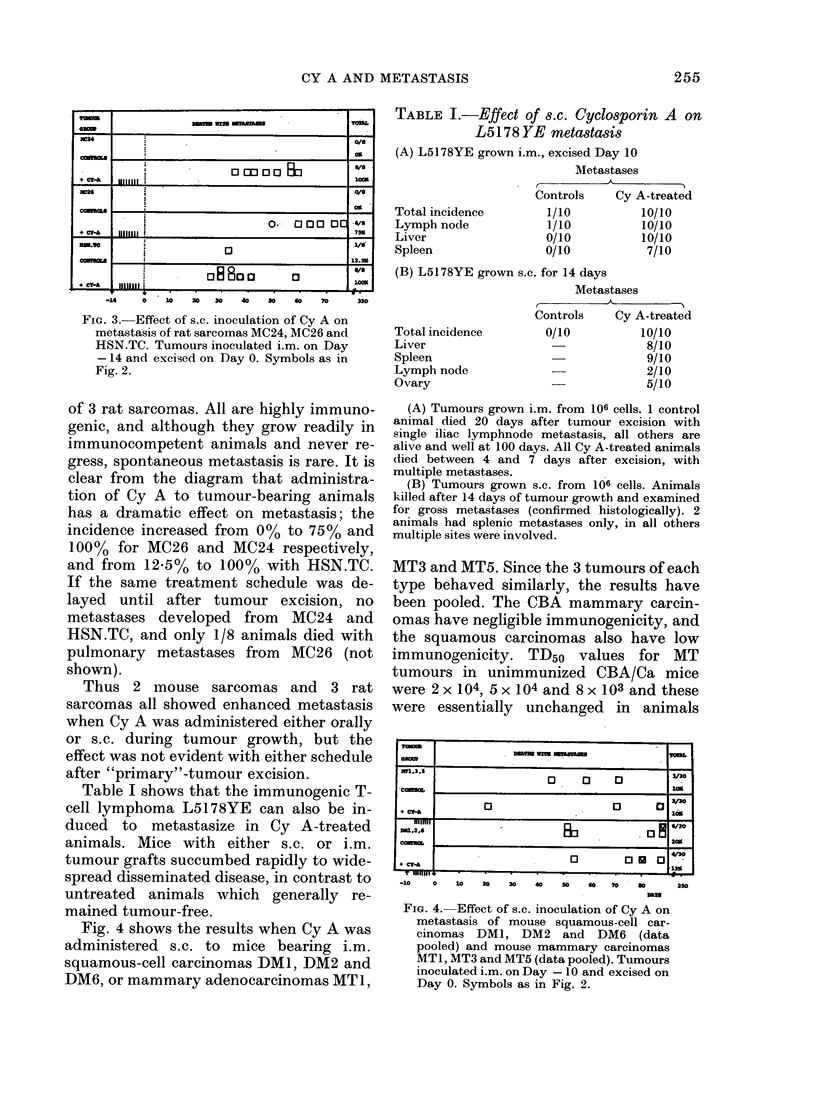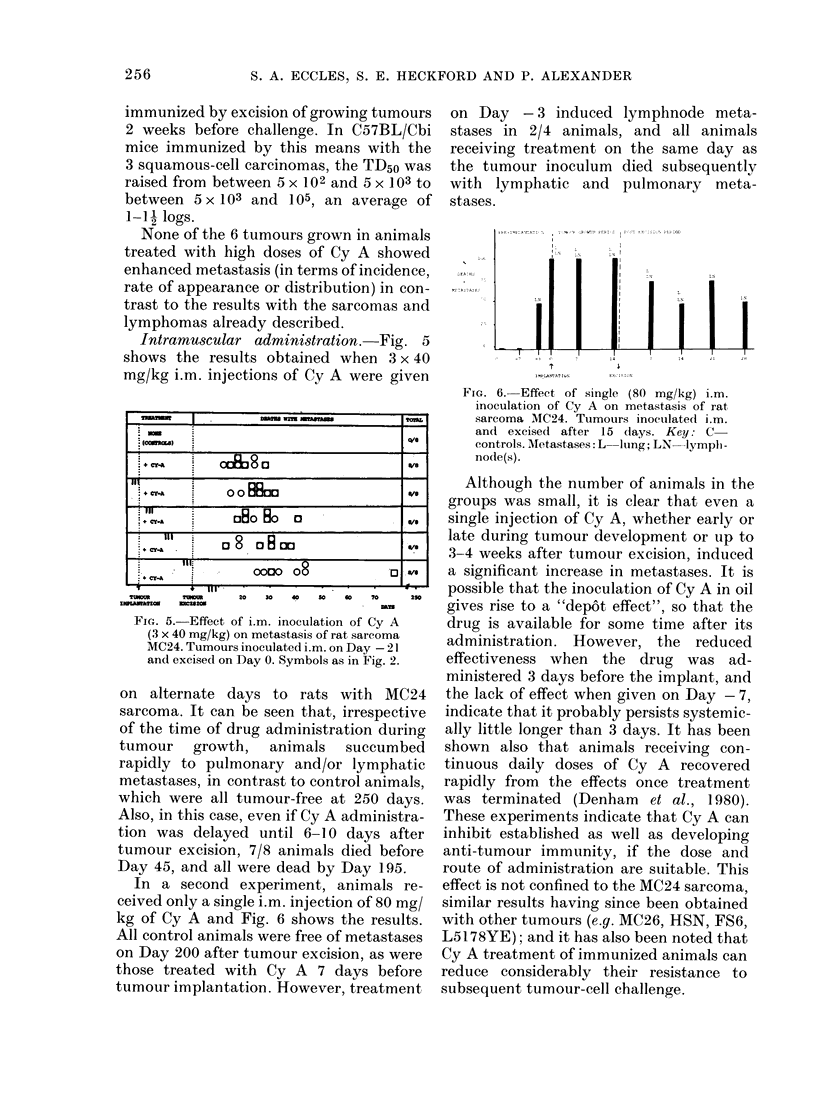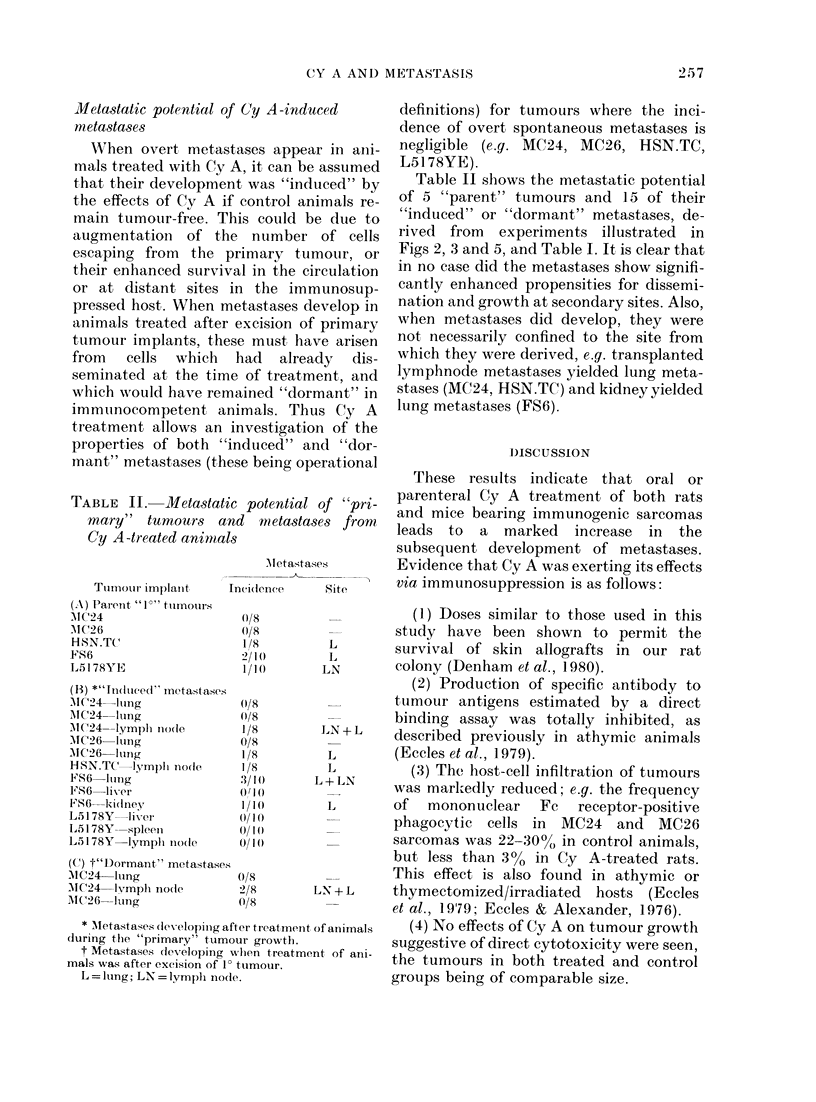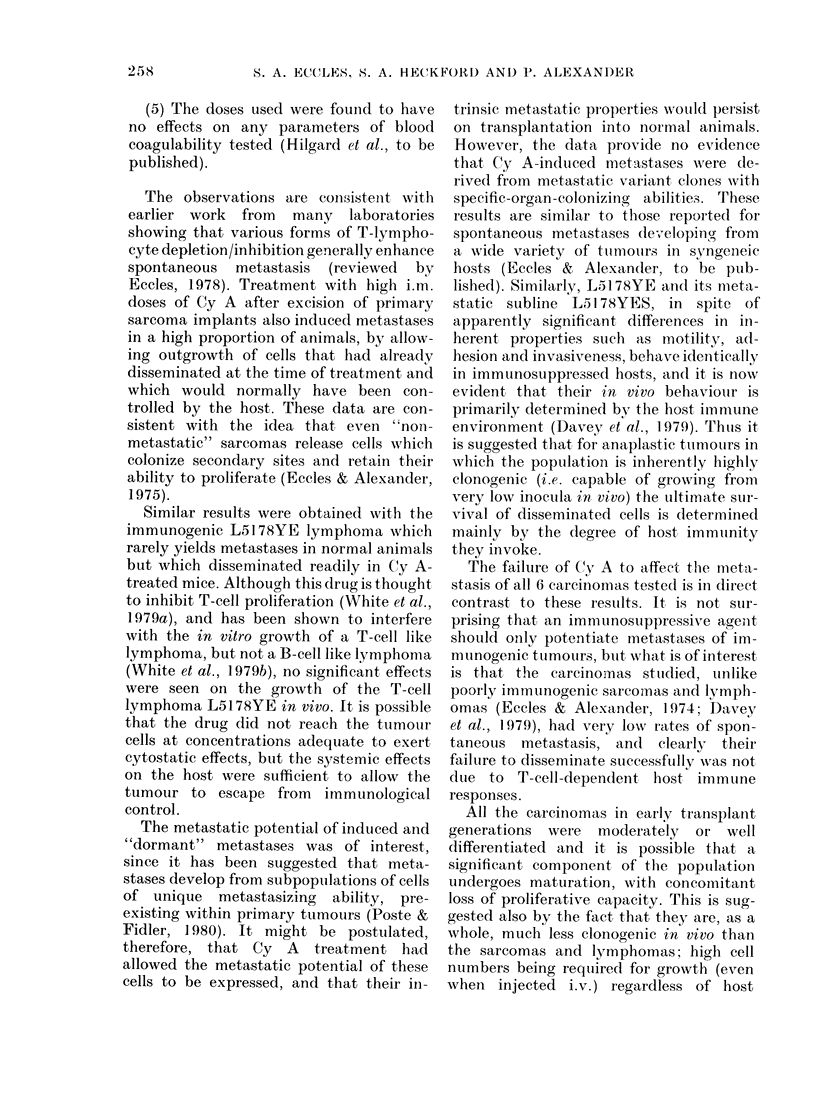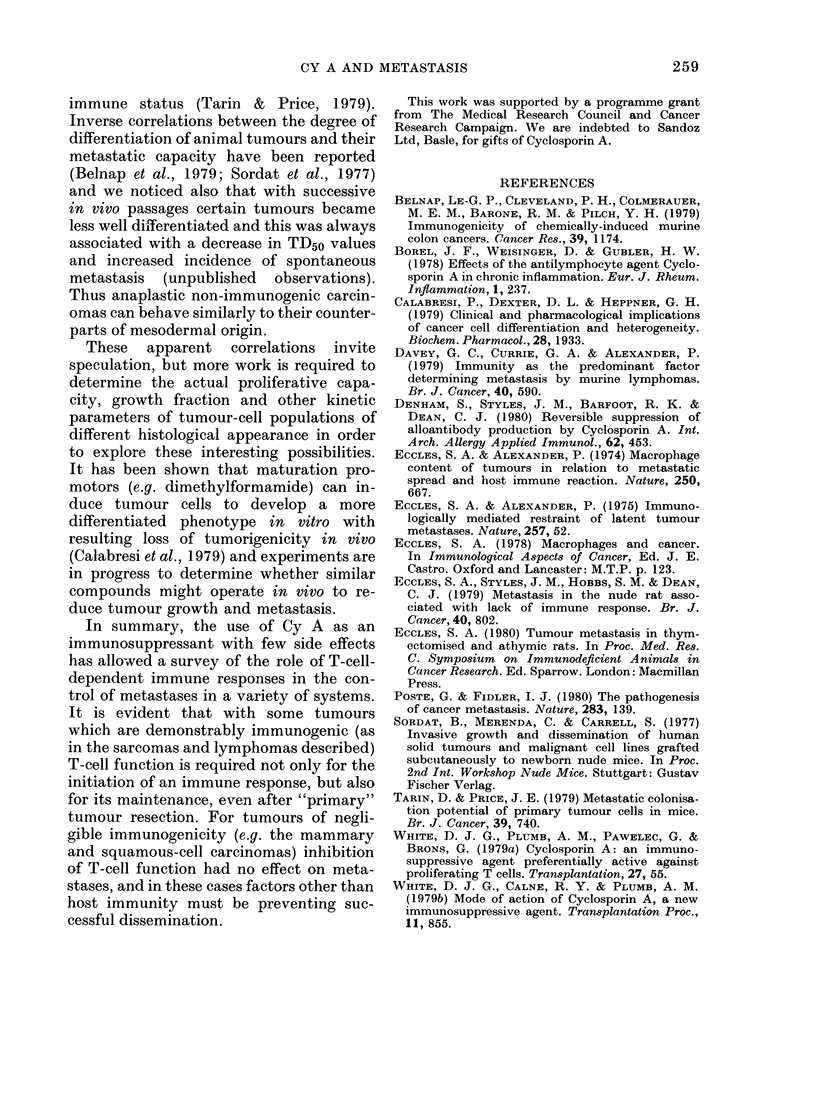# Effect of cyclosporin A on the growth and spontaneous metastasis of syngeneic animal tumours.

**DOI:** 10.1038/bjc.1980.224

**Published:** 1980-08

**Authors:** S. A. Eccles, S. E. Heckford, P. Alexander

## Abstract

Cyclosporin A (Cy A), a novel immunosuppressive agent with apparently selective inhibitory effects on T lymphocytes and little myelotoxicity, was tested for its effects on a variety of syngeneic animal tumours including sarcomas, carcinomas and a T-cell lymphoma. Cy A, given orally or parenterally in repeated doses, had no effect on the growth rates of any of the tumours tested, but a highly significant effect on metastasis was seen in many cases. All the sarcomas examined in both rats and mice, and also the lymphoma, showed a marked increase in their metastases, in some cases even when administration of Cy A was delayed until after excision of the "primary" tumour implants. In contrast no effect of Cy A on metastasis was observed in animals bearing poorly immunogenic mammary or squamous-cell carcinomas. The metastases developing in Cy A-treated animals, when transplanted into normal syngeneic animals, showed no evidence of enhanced metastatic potential compared with their "parent tumours.


*Br. J. Cancer* (1980) **42**, 252

### EFFECT OF CYCLOSPORIN A ON THE GROWTH AND SPONTANEOUS METASTASIS OF SYNGENEIC ANIMAL TUMOURS

S. A. ECCLES, S. E. HECKFORD AND P. ALEXANDER

*From the Department of Tumour Immunology, Chester Beatty Research Institute, Institute of Cancer Research, Sutton, Surrey*

Received 13 March 1980 Accepted 12 May 1980

**Summary.—Cyclosporin A (Cy A), a novel immunosuppressive agent with apparently selective inhibitory effects on T lymphocytes and little myelotoxicity, was tested for its effects on a variety of syngeneic animal tumours including sarcomas, carcinomas and a T-cell lymphoma. Cy A, given orally or parenterally in repeated doses, had no effect on the growth rates of any of the tumours tested, but a highly significant effect on metastasis was seen in many cases. All the sarcomas examined in both rats and mice, and also the lymphoma, showed a marked increase in their metastases, in some cases even when administration of Cy A was delayed until after excision of the "primary" tumour implants. In contrast no effect of Cy A on metastasis was observed in animals bearing poorly immunogenic mammary or squamous-cell carcinomas. The metastases developing in Cy A-treated animals, when transplanted into normal syngeneic animals, showed no evidence of enhanced metastatic potential compared with their"parent" tumours.**

Cyclosporin A (Cy A) is a fungal metabolite the immunosuppressive properties of which have been described by Borel *et al.* (1978) and include inhibition of humoral immunity (assayed by plaque-forming cell number and haemagglutination titre) and cell-mediated immunity (assayed by skin-graft rejection, graft-*vs*-host disease and experimental allergic encephalitis). It was shown to be effective after oral or parenteral administration, and to inhibit both primary and secondary immune responses. Its mode of action is still imperfectly understood, but in contrast to other immunosuppressive or cytostatic drugs it has little effect on haemopoietic tissues and seems preferentially to inhibit T lymphocytes at an early stage of mitogenic triggering (Borel *et al.*, 1978). Further advantages are that its effects seem to be reversed fairly rapidly, at least in some systems (Denham *et al.*, 1980) and do not seriously compromise immunity to common pathogens.

Previous work from this laboratory had shown that when animals were putatively immunosuppressed by thymectomy, whole-body X-irradiation or chronic lymph depletion, the incidence of metastases from immunogenic tumours was greatly increased and approached that of poorly immunogenic tumours (Eccles & Alexander, 1974, 1975). Although these effects could be reversed by reconstitution of the animals with lymphoid cells before tumour implantation (Eccles, 1978) the possibility that surgical stress or radiation damage had contributed to the increased incidence of metastases could not be discounted entirely. Subsequently, it was shown that similar tumours grown in congenitally athymic "nude" rats also exhibited enhanced metastasis over immune-competent hosts (Eccles *et al.*, 1979; Eccles, 1980) although the athymic rats were of mixed genetic background, and not syngeneic with the Lister Hooded/Cbi tumours under investigation. Cyclosporin A offered the possibility of studying the growth and metastasis of a wide variety of rat and mouse tumours in strictly syngeneic hosts, subjected to specific immune suppression not requiring surgery, the use of antibiotics or filterbox housing as had all previous experimental systems used. In addition, the rapidity with which Cy A exerts its effects, from which animals recover after cessation of treatment, allowed a more detailed examination of the results of suppression of host T-dependent immune responses at various stages of tumour growth and after primary-tumour excision.

### MATERIALS AND METHODS

*Rat tumours.*—Highly inbred male Lister Hooded/Cbi rats were used between 10 and 12 weeks of age. The tumours studied were induced in this colony of animals, all (except HSN) within the last 3½ years. MC24 and MC26 fibrosarcomas were induced by methylcholanthrene, and HSN sarcoma by benzo(a)-pyrene.

*Mouse tumours.*—Male, and/or female mice of inbred strains C57BL/Cbi, DBA/2 and CBA/Ca were used between 10 and 12 weeks of age. The tumours studied were as follows:

FS6 and FS29; benzpyrene-induced fibrosarcomas syngeneic with male and female C57BL/Cbi mice respectively.

DM1, DM2 and DM6; squamous-cell carcinomas induced in C57BL/Cbi mice by skin-painting with DMBA (dimethylbenzanthracene) in 1979.

MT1, MT3 and MT5; mammary adenocarcinomas arising in 1979 as a result of natural transmission of Bittner factor (mammary-tumour virus) in female CBA/Ca mice, transplanted in syngeneic virus-free female mice.

L5178YE; a methylcholanthrene-induced T-cell lymphoma of female DBA/2 mice, introduced into our Institute in 1961.

Tumours were only studied in their first 10 *in vivo* transplant generations, before fresh stocks were obtained from liquid $N_2$, and unless specified otherwise they were grown from mechanically prepared cell suspensions, and not subjected to proteolytic enzymes or *in vitro* culture.

*Cyclosporin A (Cy A).*—Cyclosporin A (OL-27-400) was kindly supplied by Sandoz Ltd, Basle, in two forms: as a powder, which was suspended in tragacanth and administered orally by intubation under light ether anaesthesia, and as a solution in oil which was given s.c. or i.m. Control animals received either oral tragacanth or inoculations of olive oil as appropriate. The dosage schedules varied and are given with the details of particular experiments.

*Tumour growth studies.*—Tumour-cell suspensions—0·05 ml (mouse) or 0·1 ml (rat)—were inoculated s.c. into the right flanks of groups of 8 or 10 syngeneic animals. Half of each group received daily s.c. injections of Cy A in the contralateral flanks, beginning on the day before tumour inoculation and continuing to the end of the experiments; controls received an equal volume of olive oil. The doses used were 20 mg/kg for rats, and 80 mg/kg for mice. Measurements of 2 perpendicular tumour diameters were made on alternate days and results expressed as the mean tumour diameter of groups of 4–5 animals. The effect of Cy A on the growth rates of mouse tumours FS6, MT1 and L5178Y and rat tumours MC24 and HSN was assessed.

*Studies of spontaneous metastasis.*—For these studies, tumours were generally grown i.m. from 0·05 ml or 0·1 ml of tumour-cell suspensions and excised after 10–14 days of growth by hind-limb amputation. Animals were then observed for up to one year, those showing symptoms of metastases were killed and necropsied, and at the end of the experiment, surviving animals were also killed and examined carefully. Suitable individual metastases were selected regularly for transplantation into further syngeneic animals, to determine whether their inherent metastatic potential was greater than in the original "primary" tumour.

(a) The effect of oral Cy A on the metastasis of mouse tumours FS6 and FS29 was studied. Ten animals received daily doses of 200 mg/kg Cy A *per os* on Days 3–10 of tumour growth; 10 were treated on Days 3–10 after tumour excision and a further 10 served as controls.

(b) The effect of s.c. administration of Cy A was studied using rat tumours MC24, MC26 and HSN.TC and mouse tumours DM1, DM2, DM6, MT1, MT3 and MT5 and L5178YE. HSN.TC refers to HSN sarcoma, freed of host cells by 3 subcultures *in vitro*; L5178YE was obtained by teasing apart spleens of tumour-bearing mice. Both these tumours were grown i.m. from $10^6$ viable cells, and L5178YE was also grown s.c. Other tumours were grown from mechanically prepared cell suspensions. Groups of 10 mice received daily doses of 80 mg/kg Cy A in oil on Days 3–10 of tumour growth, the same number of controls receiving an equivalent volume (0·02 ml) of olive oil. Groups of 8 rats —which seem to be more sensitive to Cy A— received daily doses of 20 mg/kg on Days 3–10 of tumour growth or on Days 3–10 after excision, whilst further groups of 8 controls received olive oil.

(c) The effects of i.m. Cy A and variation in the time of its administration were studied using rat sarcoma MC24. Cy A was inoculated i.m. into the non-tumour-bearing thighs of groups of 8 rats at a dose of 40 mg/kg on 3 alternate days as follows: before tumour implantation, at various times during tumour growth, and after tumour resection. Similar experiments were performed using single inoculations of 80 mg/kg Cy A.

### RESULTS

### *Growth rates of tumours*

Fig. 1 shows the growth rates of mouse sarcoma FS6 and rat sarcoma MC24 in control and Cy A-treated animals. It can be seen that daily doses of 20 or 80 mg/kg of this agent had no significant effect on either tumour; similar results were obtained with the other tumours tested. Also, tumours grown i.m. for metastasis studies were regularly dissected and weighed after excision; those from Cy A-treated and control animals were found to be of comparable mass, even when counted numbers of tumour cells were

FIG. 1.—Growth of mouse sarcoma FS6 and rat sarcoma MC24 in Cy A-treated (●) and control (■) animals.

inoculated (*e.g.* HSN.TC and L5178YE). Thus, while measurement of tumour diameter is a very crude parameter, these results indicate that Cy A is probably not directly toxic to tumour cells, and also that alterations in metastatic patterns cannot be ascribed simply to the presence of larger tumours in treated groups.

### *Influence of Cyclosporin A on patterns of spontaneous metastasis*

*Oral administration.*—Fig. 2 shows the patterns of spontaneous metastasis of mouse sarcomas FS6 and FS29, and the

FIG. 2.—Effect of orally administered Cy A on metastasis of mouse sarcomas FS6 and FS29. Tumours inoculated i.m. Day −10 and excised on Day 0. ||| days on which Cy A was given. Metastases: □ lung; ○ lymph node(s); ▽ kidney; ◇ liver.

effects of Cy A. Both tumours are immunogenic and have relatively low rates of metastasis, normally confined to the lungs, but in both cases administration of Cy A to tumour-bearing animals significantly increased the incidence and, in the case of FS6, the distribution of metastases. Cy A had no detectable effects on the incidence of metastases when given after tumour excision, although 20% of animals developed lymphnode metastases from FS29 in addition to pulmonary metastases. It is now known that Cy A is poorly absorbed when given orally in suspension, but nevertheless this regime was sufficient to enhance metastasis of both tumours.

*Subcutaneous administration.*—Fig. 3 shows the effect of Cy A on the metastasis

FIG. 3.—Effect of s.c. inoculation of Cy A on metastasis of rat sarcomas MC24, MC26 and HSN.TC. Tumours inoculated i.m. on Day −14 and excised on Day 0. Symbols as in Fig. 2.

of 3 rat sarcomas. All are highly immunogenic, and although they grow readily in immunocompetent animals and never regress, spontaneous metastasis is rare. It is clear from the diagram that administration of Cy A to tumour-bearing animals has a dramatic effect on metastasis; the incidence increased from 0% to 75% and 100% for MC26 and MC24 respectively, and from 12·5% to 100% with HSN.TC. If the same treatment schedule was delayed until after tumour excision, no metastases developed from MC24 and HSN.TC, and only 1/8 animals died with pulmonary metastases from MC26 (not shown).

Thus 2 mouse sarcomas and 3 rat sarcomas all showed enhanced metastasis when Cy A was administered either orally or s.c. during tumour growth, but the effect was not evident with either schedule after "primary"-tumour excision.

Table I shows that the immunogenic T-cell lymphoma L5178YE can also be induced to metastasize in Cy A-treated animals. Mice with either s.c. or i.m. tumour grafts succumbed rapidly to widespread disseminated disease, in contrast to untreated animals which generally remained tumour-free.

Fig. 4 shows the results when Cy A was administered s.c. to mice bearing i.m. squamous-cell carcinomas DM1, DM2 and DM6, or mammary adenocarcinomas MT1,

TABLE I.—*Effect of s.c. Cyclosporin A on L5178YE metastasis*

(A) L5178YE grown i.m., excised Day 10

| | Metastases | |
|---|---|---|
| | Controls | Cy A-treated |
| Total incidence | 1/10 | 10/10 |
| Lymph node | 1/10 | 10/10 |
| Liver | 0/10 | 10/10 |
| Spleen | 0/10 | 7/10 |

(B) L5178YE grown s.c. for 14 days

| | Metastases | |
|---|---|---|
| | Controls | Cy A-treated |
| Total incidence | 0/10 | 10/10 |
| Liver | — | 8/10 |
| Spleen | — | 9/10 |
| Lymph node | — | 2/10 |
| Ovary | — | 5/10 |

(A) Tumours grown i.m. from $10^6$ cells. 1 control animal died 20 days after tumour excision with single iliac lymphnode metastasis, all others are alive and well at 100 days. All Cy A-treated animals died between 4 and 7 days after excision, with multiple metastases.

(B) Tumours grown s.c. from $10^6$ cells. Animals killed after 14 days of tumour growth and examined for gross metastases (confirmed histologically). 2 animals had splenic metastases only, in all others multiple sites were involved.

MT3 and MT5. Since the 3 tumours of each type behaved similarly, the results have been pooled. The CBA mammary carcinomas have negligible immunogenicity, and the squamous carcinomas also have low immunogenicity. $TD_{50}$ values for MT tumours in unimmunized CBA/Ca mice were $2 \times 10^4$, $5 \times 10^4$ and $8 \times 10^3$ and these were essentially unchanged in animals

FIG. 4.—Effect of s.c. inoculation of Cy A on metastasis of mouse squamous-cell carcinomas DM1, DM2 and DM6 (data pooled) and mouse mammary carcinomas MT1, MT3 and MT5 (data pooled). Tumours inoculated i.m. on Day −10 and excised on Day 0. Symbols as in Fig. 2.

immunized by excision of growing tumours 2 weeks before challenge. In C57BL/Cbi mice immunized by this means with the 3 squamous-cell carcinomas, the $TD_{50}$ was raised from between $5 \times 10^2$ and $5 \times 10^3$ to between $5 \times 10^3$ and $10^5$, an average of $1–1\frac{1}{2}$ logs.

None of the 6 tumours grown in animals treated with high doses of Cy A showed enhanced metastasis (in terms of incidence, rate of appearance or distribution) in contrast to the results with the sarcomas and lymphomas already described.

*Intramuscular administration.*—Fig. 5 shows the results obtained when $3 \times 40$ mg/kg i.m. injections of Cy A were given

FIG. 5.—Effect of i.m. inoculation of Cy A ($3 \times 40$ mg/kg) on metastasis of rat sarcoma MC24. Tumours inoculated i.m. on Day $-21$ and excised on Day 0. Symbols as in Fig. 2.

on alternate days to rats with MC24 sarcoma. It can be seen that, irrespective of the time of drug administration during tumour growth, animals succumbed rapidly to pulmonary and/or lymphatic metastases, in contrast to control animals, which were all tumour-free at 250 days. Also, in this case, even if Cy A administration was delayed until 6–10 days after tumour excision, 7/8 animals died before Day 45, and all were dead by Day 195.

In a second experiment, animals received only a single i.m. injection of 80 mg/kg of Cy A and Fig. 6 shows the results. All control animals were free of metastases on Day 200 after tumour excision, as were those treated with Cy A 7 days before tumour implantation. However, treatment on Day $-3$ induced lymphnode metastases in 2/4 animals, and all animals receiving treatment on the same day as the tumour inoculum died subsequently with lymphatic and pulmonary metastases.

FIG. 6.—Effect of single (80 mg/kg) i.m. inoculation of Cy A on metastasis of rat sarcoma MC24. Tumours inoculated i.m. and excised after 15 days. *Key:* C—controls. Metastases: L—lung; LN—lymphnode(s).

Although the number of animals in the groups was small, it is clear that even a single injection of Cy A, whether early or late during tumour development or up to 3–4 weeks after tumour excision, induced a significant increase in metastases. It is possible that the inoculation of Cy A in oil gives rise to a "depôt effect", so that the drug is available for some time after its administration. However, the reduced effectiveness when the drug was administered 3 days before the implant, and the lack of effect when given on Day $-7$, indicate that it probably persists systemically little longer than 3 days. It has been shown also that animals receiving continuous daily doses of Cy A recovered rapidly from the effects once treatment was terminated (Denham *et al.*, 1980). These experiments indicate that Cy A can inhibit established as well as developing anti-tumour immunity, if the dose and route of administration are suitable. This effect is not confined to the MC24 sarcoma, similar results having since been obtained with other tumours (*e.g.* MC26, HSN, FS6, L5178YE); and it has also been noted that Cy A treatment of immunized animals can reduce considerably their resistance to subsequent tumour-cell challenge.

*Metastatic potential of Cy A-induced metastases*

When overt metastases appear in animals treated with Cy A, it can be assumed that their development was "induced" by the effects of Cy A if control animals remain tumour-free. This could be due to augmentation of the number of cells escaping from the primary tumour, or their enhanced survival in the circulation or at distant sites in the immunosuppressed host. When metastases develop in animals treated after excision of primary tumour implants, these must have arisen from cells which had already disseminated at the time of treatment, and which would have remained "dormant" in immunocompetent animals. Thus Cy A treatment allows an investigation of the properties of both "induced" and "dormant" metastases (these being operational definitions) for tumours where the incidence of overt spontaneous metastases is negligible (*e.g.* MC24, MC26, HSN.TC, L5178YE).

Table II shows the metastatic potential of 5 "parent" tumours and 15 of their "induced" or "dormant" metastases, derived from experiments illustrated in Figs 2, 3 and 5, and Table I. It is clear that in no case did the metastases show significantly enhanced propensities for dissemination and growth at secondary sites. Also, when metastases did develop, they were not necessarily confined to the site from which they were derived, *e.g.* transplanted lymphnode metastases yielded lung metastases (MC24, HSN.TC) and kidney yielded lung metastases (FS6).

TABLE II.—*Metastatic potential of "primary" tumours and metastases from Cy A-treated animals*

| Tumour implant | Metastases: Incidence | Metastases: Site |
|---|---|---|
| (A) Parent "1°" tumours | | |
| MC24 | 0/8 | — |
| MC26 | 0/8 | — |
| HSN.TC | 1/8 | L |
| FS6 | 2/10 | L |
| L5178YE | 1/10 | LN |
| (B) *"Induced" metastases | | |
| MC24—lung | 0/8 | — |
| MC24—lung | 0/8 | — |
| MC24—lymph node | 1/8 | LN + L |
| MC26—lung | 0/8 | — |
| MC26—lung | 1/8 | L |
| HSN.TC—lymph node | 1/8 | L |
| FS6—lung | 3/10 | L + LN |
| FS6—liver | 0/10 | — |
| FS6—kidney | 1/10 | L |
| L5178Y—liver | 0/10 | — |
| L5178Y—spleen | 0/10 | — |
| L5178Y—lymph node | 0/10 | — |
| (C) †"Dormant" metastases | | |
| MC24—lung | 0/8 | — |
| MC24—lymph node | 2/8 | LN + L |
| MC26—lung | 0/8 | — |

* Metastases developing after treatment of animals during the "primary" tumour growth.

† Metastases developing when treatment of animals was after excision of 1° tumour.

L = lung; LN = lymph node.

DISCUSSION

These results indicate that oral or parenteral Cy A treatment of both rats and mice bearing immunogenic sarcomas leads to a marked increase in the subsequent development of metastases. Evidence that Cy A was exerting its effects *via* immunosuppression is as follows:

(1) Doses similar to those used in this study have been shown to permit the survival of skin allografts in our rat colony (Denham *et al.*, 1980).

(2) Production of specific antibody to tumour antigens estimated by a direct binding assay was totally inhibited, as described previously in athymic animals (Eccles *et al.*, 1979).

(3) The host-cell infiltration of tumours was markedly reduced; *e.g.* the frequency of mononuclear Fc receptor-positive phagocytic cells in MC24 and MC26 sarcomas was 22–30% in control animals, but less than 3% in Cy A-treated rats. This effect is also found in athymic or thymectomized/irradiated hosts (Eccles *et al.*, 1979; Eccles & Alexander, 1976).

(4) No effects of Cy A on tumour growth suggestive of direct cytotoxicity were seen, the tumours in both treated and control groups being of comparable size.

(5) The doses used were found to have no effects on any parameters of blood coagulability tested (Hilgard *et al.*, to be published).

The observations are consistent with earlier work from many laboratories showing that various forms of T-lymphocyte depletion/inhibition generally enhance spontaneous metastasis (reviewed by Eccles, 1978). Treatment with high i.m. doses of Cy A after excision of primary sarcoma implants also induced metastases in a high proportion of animals, by allowing outgrowth of cells that had already disseminated at the time of treatment and which would normally have been controlled by the host. These data are consistent with the idea that even "non-metastatic" sarcomas release cells which colonize secondary sites and retain their ability to proliferate (Eccles & Alexander, 1975).

Similar results were obtained with the immunogenic L5178YE lymphoma which rarely yields metastases in normal animals but which disseminated readily in Cy A-treated mice. Although this drug is thought to inhibit T-cell proliferation (White *et al.*, 1979*a*), and has been shown to interfere with the *in vitro* growth of a T-cell like lymphoma, but not a B-cell like lymphoma (White *et al.*, 1979*b*), no significant effects were seen on the growth of the T-cell lymphoma L5178YE *in vivo*. It is possible that the drug did not reach the tumour cells at concentrations adequate to exert cytostatic effects, but the systemic effects on the host were sufficient to allow the tumour to escape from immunological control.

The metastatic potential of induced and "dormant" metastases was of interest, since it has been suggested that metastases develop from subpopulations of cells of unique metastasizing ability, pre-existing within primary tumours (Poste & Fidler, 1980). It might be postulated, therefore, that Cy A treatment had allowed the metastatic potential of these cells to be expressed, and that their intrinsic metastatic properties would persist on transplantation into normal animals. However, the data provide no evidence that Cy A-induced metastases were derived from metastatic variant clones with specific-organ-colonizing abilities. These results are similar to those reported for spontaneous metastases developing from a wide variety of tumours in syngeneic hosts (Eccles & Alexander, to be published). Similarly, L5178YE and its metastatic subline L5178YES, in spite of apparently significant differences in inherent properties such as motility, adhesion and invasiveness, behave identically in immunosuppressed hosts, and it is now evident that their *in vivo* behaviour is primarily determined by the host immune environment (Davey *et al.*, 1979). Thus it is suggested that for anaplastic tumours in which the population is inherently highly clonogenic (*i.e.* capable of growing from very low inocula *in vivo*) the ultimate survival of disseminated cells is determined mainly by the degree of host immunity they invoke.

The failure of Cy A to affect the metastasis of all 6 carcinomas tested is in direct contrast to these results. It is not surprising that an immunosuppressive agent should only potentiate metastases of immunogenic tumours, but what is of interest is that the carcinomas studied, unlike poorly immunogenic sarcomas and lymphomas (Eccles & Alexander, 1974; Davey *et al.*, 1979), had very low rates of spontaneous metastasis, and clearly their failure to disseminate successfully was not due to T-cell-dependent host immune responses.

All the carcinomas in early transplant generations were moderately or well differentiated and it is possible that a significant component of the population undergoes maturation, with concomitant loss of proliferative capacity. This is suggested also by the fact that they are, as a whole, much less clonogenic *in vivo* than the sarcomas and lymphomas; high cell numbers being required for growth (even when injected i.v.) regardless of host immune status (Tarin & Price, 1979). Inverse correlations between the degree of differentiation of animal tumours and their metastatic capacity have been reported (Belnap *et al.*, 1979; Sordat *et al.*, 1977) and we noticed also that with successive *in vivo* passages certain tumours became less well differentiated and this was always associated with a decrease in $TD_{50}$ values and increased incidence of spontaneous metastasis (unpublished observations). Thus anaplastic non-immunogenic carcinomas can behave similarly to their counterparts of mesodermal origin.

These apparent correlations invite speculation, but more work is required to determine the actual proliferative capacity, growth fraction and other kinetic parameters of tumour-cell populations of different histological appearance in order to explore these interesting possibilities. It has been shown that maturation promotors (*e.g.* dimethylformamide) can induce tumour cells to develop a more differentiated phenotype *in vitro* with resulting loss of tumorigenicity *in vivo* (Calabresi *et al.*, 1979) and experiments are in progress to determine whether similar compounds might operate *in vivo* to reduce tumour growth and metastasis.

In summary, the use of Cy A as an immunosuppressant with few side effects has allowed a survey of the role of T-cell-dependent immune responses in the control of metastases in a variety of systems. It is evident that with some tumours which are demonstrably immunogenic (as in the sarcomas and lymphomas described) T-cell function is required not only for the initiation of an immune response, but also for its maintenance, even after "primary" tumour resection. For tumours of negligible immunogenicity (*e.g.* the mammary and squamous-cell carcinomas) inhibition of T-cell function had no effect on metastases, and in these cases factors other than host immunity must be preventing successful dissemination.

This work was supported by a programme grant from The Medical Research Council and Cancer Research Campaign. We are indebted to Sandoz Ltd, Basle, for gifts of Cyclosporin A.

### REFERENCES

Belnap, Le-G. P., Cleveland, P. H., Colmerauer, M. E. M., Barone, R. M. & Pilch, Y. H. (1979) Immunogenicity of chemically-induced murine colon cancers. *Cancer Res.*, **39**, 1174.

Borel, J. F., Weisinger, D. & Gubler, H. W. (1978) Effects of the antilymphocyte agent Cyclosporin A in chronic inflammation. *Eur. J. Rheum. Inflammation*, **1**, 237.

Calabresi, P., Dexter, D. L. & Heppner, G. H. (1979) Clinical and pharmacological implications of cancer cell differentiation and heterogeneity. *Biochem. Pharmacol.*, **28**, 1933.

Davey, G. C., Currie, G. A. & Alexander, P. (1979) Immunity as the predominant factor determining metastasis by murine lymphomas. *Br. J. Cancer*, **40**, 590.

Denham, S., Styles, J. M., Barfoot, R. K. & Dean, C. J. (1980) Reversible suppression of alloantibody production by Cyclosporin A. *Int. Arch. Allergy Applied Immunol.*, **62**, 453.

Eccles, S. A. & Alexander, P. (1974) Macrophage content of tumours in relation to metastatic spread and host immune reaction. *Nature*, **250**, 667.

Eccles, S. A. & Alexander, P. (1975) Immunologically mediated restraint of latent tumour metastases. *Nature*, **257**, 52.

Eccles, S. A. (1978) Macrophages and cancer. In *Immunological Aspects of Cancer*, Ed. J. E. Castro. Oxford and Lancaster: M.T.P. p. 123.

Eccles, S. A., Styles, J. M., Hobbs, S. M. & Dean, C. J. (1979) Metastasis in the nude rat associated with lack of immune response. *Br. J. Cancer*, **40**, 802.

Eccles, S. A. (1980) Tumour metastasis in thymectomised and athymic rats. In *Proc. Med. Res. C. Symposium on Immunodeficient Animals in Cancer Research*. Ed. Sparrow. London: Macmillan Press.

Poste, G. & Fidler, I. J. (1980) The pathogenesis of cancer metastasis. *Nature*, **283**, 139.

Sordat, B., Merenda, C. & Carrell, S. (1977) Invasive growth and dissemination of human solid tumours and malignant cell lines grafted subcutaneously to newborn nude mice. In *Proc. 2nd Int. Workshop Nude Mice*. Stuttgart: Gustav Fischer Verlag.

Tarin, D. & Price, J. E. (1979) Metastatic colonisation potential of primary tumour cells in mice. *Br. J. Cancer*, **39**, 740.

White, D. J. G., Plumb, A. M., Pawelec, G. & Brons, G. (1979*a*) Cyclosporin A: an immunosuppressive agent preferentially active against proliferating T cells. *Transplantation*, **27**, 55.

White, D. J. G., Calne, R. Y. & Plumb, A. M. (1979*b*) Mode of action of Cyclosporin A, a new immunosuppressive agent. *Transplantation Proc.*, **11**, 855.